# Publishing protocols for trials of complex interventions before trial completion – potential pitfalls, solutions and the need for public debate

**DOI:** 10.1186/s13063-016-1757-7

**Published:** 2017-01-09

**Authors:** Anna Purna Basu, Janice Elizabeth Pearse, Tim Rapley

**Affiliations:** 1 Institute of Neuroscience, Newcastle University, Newcastle upon Tyne, NE1 7RU UK; 2 Department of Paediatric Neurology, Newcastle upon Tyne Hospitals NHS Foundation Trust, Newcastle upon Tyne, NE7 7DN UK; 3 Therapy Services, Newcastle upon Tyne Hospitals NHS Foundation Trust, Newcastle upon Tyne, NE7 7DN UK; 4 Institute of Health and Society, Newcastle University, Newcastle upon Tyne, NE2 4AX UK

**Keywords:** Open Science, Complex interventions, Protocols, Behaviour change, Therapy interventions, Clinical trials, E-Health, Advance publication, Trial registration, Codesign

## Abstract

**Background:**

Open Science is ‘the movement to make scientific research, data and dissemination accessible to all levels of an inquiring society’.

In the spirit of the Open Science movement, *advance* publication of protocols for clinical trials is now being advocated by BioMed Central, BMJ Open and others. Simultaneously, participants are becoming increasingly active in their pursuit and sharing of trial- and health- related information. Whilst access to protocols alongside published trial findings has clear benefits, advance publication of trial protocols is potentially problematic for trials of complex behavioural interventions. In this article we explain, with examples, how this could lead to unblinding, ‘contamination’ between intervention and control groups and deliberate biasing of assessment outcomes by participants. We discuss potential solutions and demonstrate the need for public debate about how this issue is best managed.

**Conclusion:**

Triallists may still be underestimating participants’ interest in information. This needs to change: joint and open discussions with the public are needed to inform how we should proceed.

## Background

Open Science is ‘the movement to make scientific research, data and dissemination accessible to all levels of an inquiring society’ [1]. In the spirit of the Open Science movement, *advance* publication of protocols for clinical trials is now being advocated by BioMed Central, BMJ Open and others. Simultaneously, participants are becoming increasingly active in their pursuit and sharing of trial- and health-related information. For the vast majority of trials this is a good thing: access to well-written protocols alongside published trial findings has clear benefits [2, 3], including the provision of more information than that available in trial registries. Advance publication of protocols is also about transparency so that post-trial publications are consistent (in process and analysis) or have to have account for protocol deviations.

A good protocol must specify all interventions in detail, so that they are replicable [4]. For novel or expensive drug interventions or those involving devices, lack of access to the intervention outside the trial arm limits the possibility of contamination even if the details are fully available in the protocol. However, a published protocol makes other interventions, such as a fully described therapy or self-administered lifestyle change, accessible outside of the trial context. The resulting problems could include unblinding, ‘contamination’ between intervention and control groups and deliberate biasing of assessment outcomes by participants. We discuss examples and demonstrate the need for public debate about how this issue is best managed.

### Potential for unblinding and contamination

An interesting example of the potential difficulties faced with published protocols is that of Eliasson et al. [5]. Their paper sets out the protocol for a randomised controlled trial comparing a form of modified constraint therapy (baby-CIMT) versus massage for infants at risk of developing unilateral cerebral palsy. Both approaches are described in detail. The hypotheses are that the baby-CIMT group will develop manual ability in the involved hand faster than the massage group and that these differences will remain at the age of 2 years; additionally, that parents in the baby-CIMT group will feel more competent about parenting. The protocol also states that parents will be blinded to the study hypotheses but not to the group allocation – clearly this will not be the case for parents who have accessed the published protocol, which may alter their engagement with the trial. This could be a particular problem for participants who discover in this way that they are in the control group. Parents may be provoked to change their behaviour and seek the active intervention for their child, given that clinical benefit is a major item on parental agendas when entering trials [6–8]. Unblinding and contamination due to patients allocated to placebo becoming aware of the nature of a therapy intervention being received by other nearby patients is an ongoing problem [9]. Though we are not aware of examples where this has occurred due to patients accessing the published protocol, it is a theoretical risk. Furthermore, parents of newborn infants allocated to the control group in a recent unblinded randomised controlled trial showed an ‘almost universal experience of disappointment’, with a few admitting either an attempt or success in obtaining the treatment outside of the trial context [10]. This latter behaviour has been described as ‘compensatory rivalry’ by Cook and Campbell [11].

We contacted the authors of the baby-CIMT paper [5]; they were not aware of any parents having accessed the online protocol but this was not a focus of their investigation. Interestingly, the protocol was published relatively late into the data collection period (trial start date September 2012; protocol publication date June 2014; study completion date December 2015); this may have been a protective factor.

### Biasing of assessment outcomes

Access to the published protocol also gives participants the opportunity to prepare for assessments in order to influence their outcome. We first identified this as a genuine risk when we undertook a focus group to discuss the details of a planned pilot feasibility study. One of the suggested questionnaires (Parenting Sense of Competence Scale [12]) required the parent to indicate on a five-point scale the extent to which they agreed or disagreed with each of a series of statements. One parent commented that it reminded her of her experiences with the Edinburgh Postnatal Depression Scale [13] after she had had her second child. Specifically, she had ‘Googled’ the scale and prepared her answers prior to her visit from the health visitor in order to avoid being identified as having possible depression (see Table 1). She was not the only parent who commented that they might give misleading responses to questions in order to avoid exposing vulnerabilities. Whilst not singled out by the Cochrane Collaboration as a specific form of bias [14], we think that manipulation of assessment outcomes by participants merits further investigation. The Cochrane Collaboration describes detection bias, in which there are differences between the intervention and control groups with respect to outcome assessment due to knowledge of the assessor about treatment assignment. We describe a different situation, in which knowledge of the nature of the assessment by the participant could lead to a deliberate alteration of their response. In essence it represents a potential form of response bias [15], i.e. ‘a systematic tendency to respond to a range of questionnaire items on some basis other than the specific item content’.

**Table 1:** Biassing of assessment outcomes: example from a codesign workshop

Emma: ‘To be honest, like, I, from my experience, and I had a really hard time after Edgar was born, my second one. And, erm, had to fill in Edinburgh thing.’	
Anna: ‘Yeah, the depression thing, yeah.’	
Emma: ‘Yeah, and I totally, like, filled it in ‘cause I didn’t want to have depression (laughter), so like, I like, I Googled it and everything, you know, to see what score you needed. (Laughter) and, erm.‘	
Anna: ‘Wow.’	
Emma: ‘Well no, I Goo- ‘cause you can do it online can’t you? And then I came up with like 12.5 or something and you needed 11 to not have depression, so I was like… And then the health visitor came round so I was thinking, “Right, 11” …’	
(Participant names have been changed to respect confidentiality)	

### Is this really a new problem?

Some of the issues we describe have been seen in other contexts for a long time. It comes as no surprise that study participants might alter behaviours which they know or suspect are being monitored. In the field of experimental psychology, such effects are covered by the term ‘demand characteristics’, defined as ‘the totality of cues and mutual expectations which inhere in a social context … which serve to influence the behaviour and/or self-reported experience of the research receiver’ [16]; though caution has been advocated in using this term outwith the laboratory setting [17], with the broader term ‘research participation effects’ preferred [18]. What is new is the extent to which these problems scale up in the shift towards increasingly accessible information, increasingly early in the timeline of ongoing research. It is now easy to seek out detailed information on the nature of many assessment tools on the Internet, potentially influencing performance in those assessments.

### How big could the problem be?

It is easy to look up a study protocol; many are published in open access journals and are freely available. In 2015 over 80% of the developed world (and over one third of the developing world) had Internet access at home [19]. Use of the Internet to obtain health-related information is now the norm [20], so there is nothing to stop participants from accessing published protocols online. Such information is likely to be shared more widely through the use of social media [21] and online patient communities [22]. Information about previous similar trials (either from academic journals or reports in the news or social media) may also influence the understanding and behaviour of participants.

Participants can potentially determine which trial arm they are in, and choose to follow instructions related to items from any trial arm (or none), based on their understanding of the stated hypotheses. Whilst Information Sheets could also prompt similar behaviours, the information in the published protocol is more detailed, yet often appears to be written with the assumption that it will not be read by participants. This adds to the problem because there is an additional risk that the accessed information may be misinterpreted in a wide range of ways. However, we simply do not know the degree to which these factors currently impact on clinical trials of behavioural change interventions. This compounds the existing problem that adherence to behavioural change interventions is often low even in the absence of other considerations [23].

To declare that advance publication of protocols for certain trial types could bias outcomes may seem a step in the wrong direction, but to deny the potential problem is foolish. At the moment, the cast of characters who might engage with published protocols is still seen as editors, funders, researchers and reviewers, all actively involved for good scientific reasons. Two other more opaque figures remain, namely readers and patients. Currently, patients are configured as users of this information in a specific way, as ‘prospective volunteers’ seeking information about potential trials to take part in. If we also see them as part of the category of reader, they may also be ‘citizen scientists’ who are now empowered to discovering deviations in protocols. However, they can also engage with this information in alternate ways, as ‘participants in trials’ or as relatives or friends of those taking part in trials. This last form of engagement is in some ways the most problematic for ongoing trials. If the patients are prospective volunteers then the protocol could supplement the Information Sheet and help them to decide whether to enter the trial. It could also influence their conduct when they enter the trial. If, instead, they are already taking part in the trial and subsequently access the protocol and change their behaviour, the trial data will be affected. Participants could also be concerned or even angry that certain information was withheld. Maximising the quality of the Information Sheets provided to participants may avoid this to some extent. However, with full transparency it becomes impossible to undertake randomised controlled trials with blinding of participants for certain types of complex intervention.

### A way forward?

It would be interesting to ask participants to what extent they or their significant others sought information regarding their trial beyond that provided by the research team, what they gleaned and whether it influenced their behaviour in relation to the trial. Assuming that participants have good memories and a willingness to share such information, this could help us to understand the current scale of the problem.

If published protocols are found to influence the behaviour of participants in complex intervention trials, trial preregistration with embargoed preregistration of the detailed protocol until trial completion may be the best option. This solution is already available – protocols registered on the Open Science Framework can be embargoed for up to 4 years before they are published. The embargo limit is key in avoiding publication bias or even covert registration of multiple protocols.

Another potential solution is the Registered Reports initiative [24]. This provides the opportunity to submit a detailed research proposal to a journal for peer review, including methods and planned analyses, prior to commencing the research. The journal can then provisionally agree in advance to publish the final study. This practice could encourage high standards of research integrity whilst keeping the protocol under wraps until the study is complete.

Trial preregistration, that requires declaration of the primary outcome measure in advance, has probably contributed to a recent increase in reported null effects in clinical trials [25] and could also help reduce duplication of research efforts even without a full published protocol. The complex intervention descriptions on trial preregistration websites probably lack the detail needed to allow replication of trial behaviours – but the problems of unblinding and biasing of assessment outcomes could still occur. Trial preregistration also helps protect the integrity of published research: one of the authors (AB) has had experience of discovering serious unexplained deviations from the protocol in a submitted paper based on trial registration details alone. However, published reports of trials have not infrequently deviated in significant and unexplained ways from published registry entries, though similar deviations from published protocols are also reported [26, 27].

What advantages of advance protocol publication do we risk losing in this field? Looking at the statement from BMJ Open [28], advance publication is about ‘enabling researchers and funding bodies to stay up to date in their fields’, preventing duplication of work and facilitating collaboration. Arguably, trial preregistration data would suffice for these purposes, whilst reducing the contamination risk and still allowing potential participants to search for studies for which they may be eligible. Alternatively, published protocols will have to be written with the expectation that they may be accessed by participants (or members of their social circle), in which case we can expect to encounter the problems described above, making it extremely hard or impossible to undertake certain types of trial. Given the calls for more comprehensive reporting of all the stages of the development of complex interventions [29] alongside the development of reporting guidelines for these stages [30], similar problems can be predicted with papers describing the development of the content and delivery of interventions.

The onus is now on researchers to smarten up experimental designs to deal with these potential problems. Objective tracking of adherence, for example, through smartphone data, is one approach. Researchers could also draw up contracts with participants whereby they agree not to attempt to unblind themselves through the use of the Internet or social media. This approach could of course backfire by providing a more explicit list of unblinding options for those hungry for information.

## Conclusions

The Open Science movement has emerged alongside the rise of more active, engaged, patients and is here to stay. It has furthered the drive for increased research transparency which is overall extremely beneficial. As part of this drive, advanced publication of trial protocols is advocated, with a full description of interventions. This causes difficulties for trials of complex behavioural change interventions, such as unblinding, contamination and biasing of questionnaire-based assessments. Triallists may still be underestimating the public, and especially participants’ interest in information [22]. This needs to change: joint and open discussions with the public are needed to inform how we should proceed as there is currently no optimal solution.
